# In silico analysis and homology modeling of strictosidine synthase involved in alkaloid biosynthesis in *catharanthus roseus*

**DOI:** 10.1186/s43141-020-00049-3

**Published:** 2020-08-28

**Authors:** Archna Sahay, Anil Piprodhe, Mashitha Pise

**Affiliations:** 1 Department of Biochemistry, Hislop College, Temple Road, Civil Lines, Nagpur, Maharashtra 440001 India; 2 Dr. D Y Patil ACS College, Pimpri, Pune, Maharashtra 411018 India

**Keywords:** *Catharanthus roseus*, STR, SOSUI, PROCHECK, PROVE

## Abstract

**Background:**

In this study, strictosidine synthase (STR) from *Catharanthus roseus* that plays an important role in alkaloid biosynthesis was selected. The purpose of this work was to perform in silico analysis and to predict the three-dimensional structure of this protein that is not available.

**Results:**

Physicochemical characterization was performed by Expasy’s Protparam server. The computed theoretical isoelectric point (pI) found to be less than 7 indicates the acidic nature of this protein. The aliphatic index 73.04 indicates the thermal stability of the protein. Grand average hydropathy (GRAVY) was predicted to be − 285; this lower value of GRAVY shows the possibility of better interaction of this protein with water. Functional analysis of these proteins was performed by SOSUI server which predicted the transmembrane helix. Secondary structure analysis was carried out by SOPMA that revealed that Alpha helix dominated among secondary structure elements followed by random coil, extended strand, and beta turns. The modeling of the three-dimensional structure of the STR was performed by Swiss model. The model was validated using protein structure checking tools PROCHECK and PROVE.

**Conclusions:**

This study reveals in silico analysis by Expasy Protparam server, SOPMA, and SOSUI server. Homology modeling of STR was performed by Swiss model.

## Background

*Catharanthus roseus* (*C. roseus*) is an important medicinal plant used to treat various diseases. The plant is distributed throughout the world. It is known to produce modern chemotherapeutic agent for pain-relieving properties [1]. One of the important types of alkaloid is the vinblastine produced from *C. roseus* which was reported due to its antitumor activity and has wide pharmaceutical use [2]. It has been reported that accumulation of free radicals can cause pathological conditions such as ischemia, asthma, arthritis, inflammation, neurodegeneration, Parkinson’s diseases, mongolism, aging process, and perhaps dementia [3]. The flower petals, seeds, and other parts of *Catharanthus roseus* exhibit antioxidant properties. Thus, phenolic compounds have redox properties that act as reducing agents, hydrogen donors, singlet oxygen quenchers, or metal chelator.

*C. roseus* was of enormous pharmaceutical interest because it contains more than 120 terpenoid indole alkaloids (TIAs). Terpenoid indole alkaloids (TIAs) are among the most important secondary metabolites in plants that play important roles in the growth and reproductive development of plants [4]. Over 100 different TIAs were discovered in *Catharanthus roseus* (periwinkle) [5]. Strictosidine was found to be the key biosynthetic precursor of TIA. It was presented in a wide variety of higher plants [6]. This important molecule is generated by strictosidine synthase (STR) from tryptamine and secologanin and catalyzes the synthesis of 3-a(S)-strictosidine. STR 1 is the first STR gene isolated from *Rauvolfia serpentina*. STR1 catalyzes the Pictet-Spengler reaction between tryptamine and secologanin and is a key enzyme for the biosynthesis of alkaloids [7, 8]. The Pictet-Spengler reaction was a two-part reaction. First, an electron-rich aromatic amine and an aldehyde condense to form an iminium species. Second, an electrophilic aromatic substitution reaction occurs in which the aryl amine attacks the electrophilic iminium to yield a positively charged intermediate which is then deprotonated to yield the β-carboline product(s). In nonenzymatically catalyzed reactions, two enantiomers are typically formed, but strictosidine synthase catalyzes the asymmetric synthesis of the strictosidine 3 diastereomer [9].

This was the central reaction in the biosynthesis of the entire family of monoterpenoid indole alkaloids in plants that includes vincamine, ajmaline, raubasine, quinine, vinblastine, reserpine, vincamine, C-toxiferen I, and camptothesine. STR proteins are involved in different physiological and biochemical pathways. The monoterpenoid-derived indole alkaloids were one of the structurally largest and pharmacologically most diverse alkaloid families in higher plants. These alkaloids have medical applications and therapeutics include the treatment of cancer (vinblastine or the camptothecin-derivative topotecan), malaria (quinine), hypertension (raubasine and reserpine), schizophrenia (reserpine in high dosage), disturbed cerebral blood flow (vincamine), and antiarrhythmic heart disorders (ajmaline), from the Indian medicinal plant *Rauvolfia serpentina* [10].

The major drawbacks of experimental mehod used to charecterize protein was involvement of high cost and time consumption. Thus, these methods were not amenable to high throughput techniques. In silico approaches provide a viable solution to these problems. Computational tools provide researchers to understand physicochemical and structural properties of proteins. The amino acid sequence provides most of the information required for determining and characterizing the molecule’s function and physical and chemical properties. In this study, the in silico analysis and homology modeling studies on STR involved in alkaloid biosynthesis was reported. The three-dimensional structure for this protein was yet not available. Hence to describe its structural features and to understand molecular function, the model structures for this protein was constructed. Physicochemical characterization was performed by computing theoretical isoelectric point (pI), molecular weight, total number of positive and negative residues, extinction coefficient, instability index, aliphatic index, and grand average hydropathy (GRAVY). Functional analysis and secondary structure prediction of these proteins was performed by SOSUI server and SOPMA, respectively. The modeling of the three-dimensional structure of the proteins was performed by Swiss model. The model was validated using protein structure checking tools PROCHECK and PROVE.

## Methods

### Ethics approval

This article does not contain any studies with human participants or animals performed by any of the authors.

### Sequence retrieval

Sequences of proteins involved in alkaloid biosynthesis of *C. roseus* were retrieved from the SWISSPROT, a public domain protein database [11]. Table 1 shows the protein sequences considered in this study. The protein sequences were retrieved in FASTA format and used for further analysis.

**Table 1:** Protein sequence considered for the study

Protein	Accession number	Length
STR	P18417	352

### Physicochemical characterization

For physicochemical characterization, theoretical pI, molecular weight, total number of positive and negative residues, extinction coefficient [12], instability index [13], aliphatic index [14], and grand average hydropathy (GRAVY) [15] were computed using the Expasy’s ProtParam server [16]. The results were shown in Table 2.

**Table 2:** Parameters computed using Expasy’s Protparam

Protein	Strictosidine synthase
Accession number	P18417
Sequence length	352
M. wt	39093.72
pl	5.14
II	47.42
AI	73.04
GRAVY	− 0.285

### Functional characterization

The SOSUI server performed the identification of transmembrane regions. Table 3 represents the transmembrane region identified for these proteins. Prosite is a database of protein families and domains [17]. Table 4 represents the output of Prosite that was recorded in terms of the length of amino residues of protein with specific profiles and patterns.

**Table 3:** Transmembrane regions identified by SOSUI server

Protein	Accession No.	Types	Transmembrane helix
STR	P18417	Membrane protein	One transmembrane helix

**Table 4:** Functional characterization of proteins of *C. roseus* at Prosite

Proteins	Accession number	Motif found	Position in the protein
STR	P18417	NO hits	

### Secondary structure prediction

SOPMA [18] was employed for calculating the secondary structural features of the selected protein sequences considered for this study. The results were presented in Table 5.

**Table 5:** Secondary structure prediction by SOPMA SERVER

Proteins secondary structure	P18417
Alpha helix	15.91%
310 helix	0.00%
Pi helix	0.00%
Beta bridge	0.00%
Extended strand	34.09%
Beta turn	12.78%
Bend region	0.00%
Random coil	37.22%
Ambiguous states	0.00%
Other states	0.00%

### Model building and evaluation

The modeling of the three-dimensional structure of the proteins was performed by Swiss model [19]. The overall stereochemical property of the protein was assessed by Ramachandran plot analysis [20]. The validation for structure models obtained from the two software tools was performed by using PROCHECK [21]. The models were further checked with PROVE. The results of PROCHECK and PROVE analysis was shown in Tables 6 and 7, respectively. Structural analysis was performed and figures representations were generated with Swiss PDB Viewer [22].

**Table 6:** Ramachandran plot calculation and analysis of the models from Swiss model and computed with the PROCHECK program

Server	Proteins	Accession number	Regions	%
Swiss model	STR	P18417	Residues in the most favored region	88.8
			Residues in additionally allowed region	10.9
			Residues in generously allowed region	0.4
			Residues in disallowed region	0.0

**Table 7:** PROVE PLOT values for STR

Protein	Accession number	Information	Values
Strictosidine synthase	P18417	*Z* score mean	0.501
		*Z* score std dev	1.269
		*Z* score RMS	1.364

## Results

In this study, STR involved in the first step of alkaloid biosynthesis present in *C. roseus* was selected. Sequence was retrieved from UNIPROT in FASTA format and used for further analysis. Table 1 shows the protein sequences considered.

Expasy’s Protparam tool was used to compute parameters to analyze physicochemical properties of this protein. The results were shown in Table 2. The computed isoelectric point value of P18417 was less than 7 (pI < 7). The instability index value for P18417 was found to be 47.42. Aliphatic index for the selected protein sequence was found to be 73.04. The grand average hydropathy (GRAVY) was found to be − 285. SOSUI is a functional analysis tool which distinguishes between membrane and soluble proteins from amino acid sequences and predicts the transmembrane helices. In Table 3, transmembrane regions and their length were tabulated. The functions of alkaloid proteins of *C. roseus* were analyzed by submitting the amino acid sequence to Pro site server. The result was presented in Table 4. The secondary structure of STR was predicted by SOPMA (Self-Optimized Prediction Method with Alignment) which correctly predicts 69.5% of amino acids for a state description of the secondary structure prediction [16]. Table 5 shows the results obtained by SOPMA. The secondary structure was predicted by using default parameters (window width, 17; similarity threshold, 8; and number of states, 4).

### Homology modeling and validation of structure of STR

**Fig. 1:**
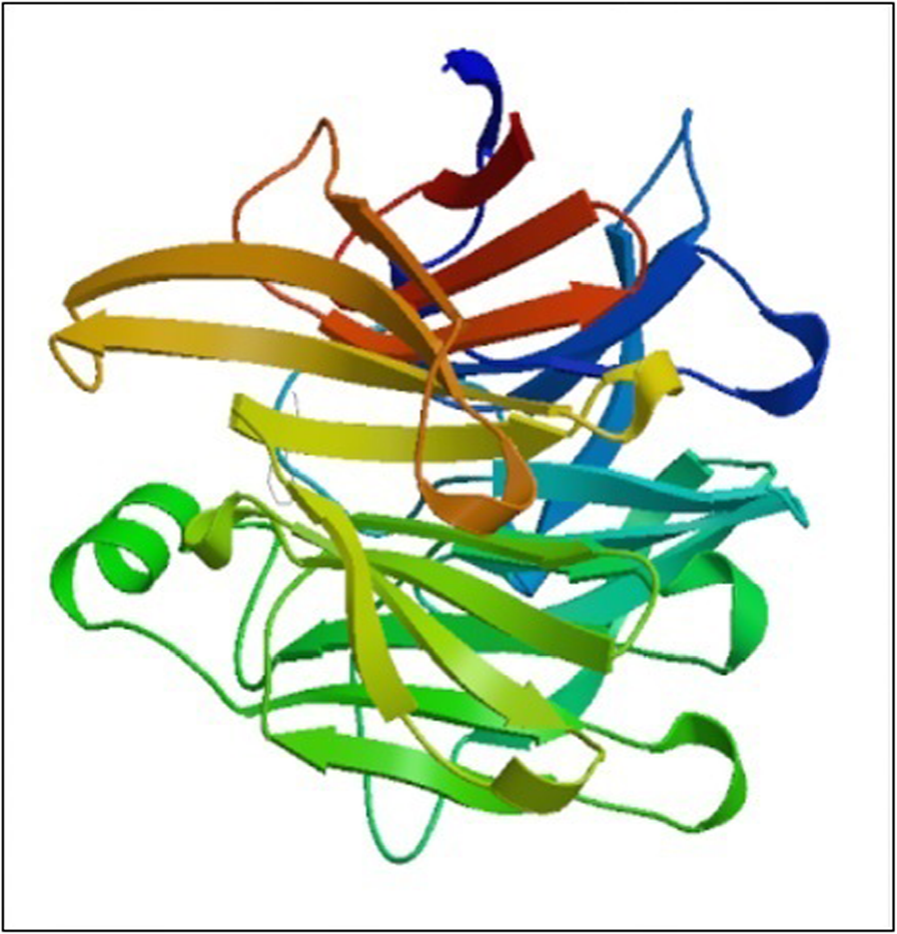
Predicted 3D structure of STR

**Fig. 2:**
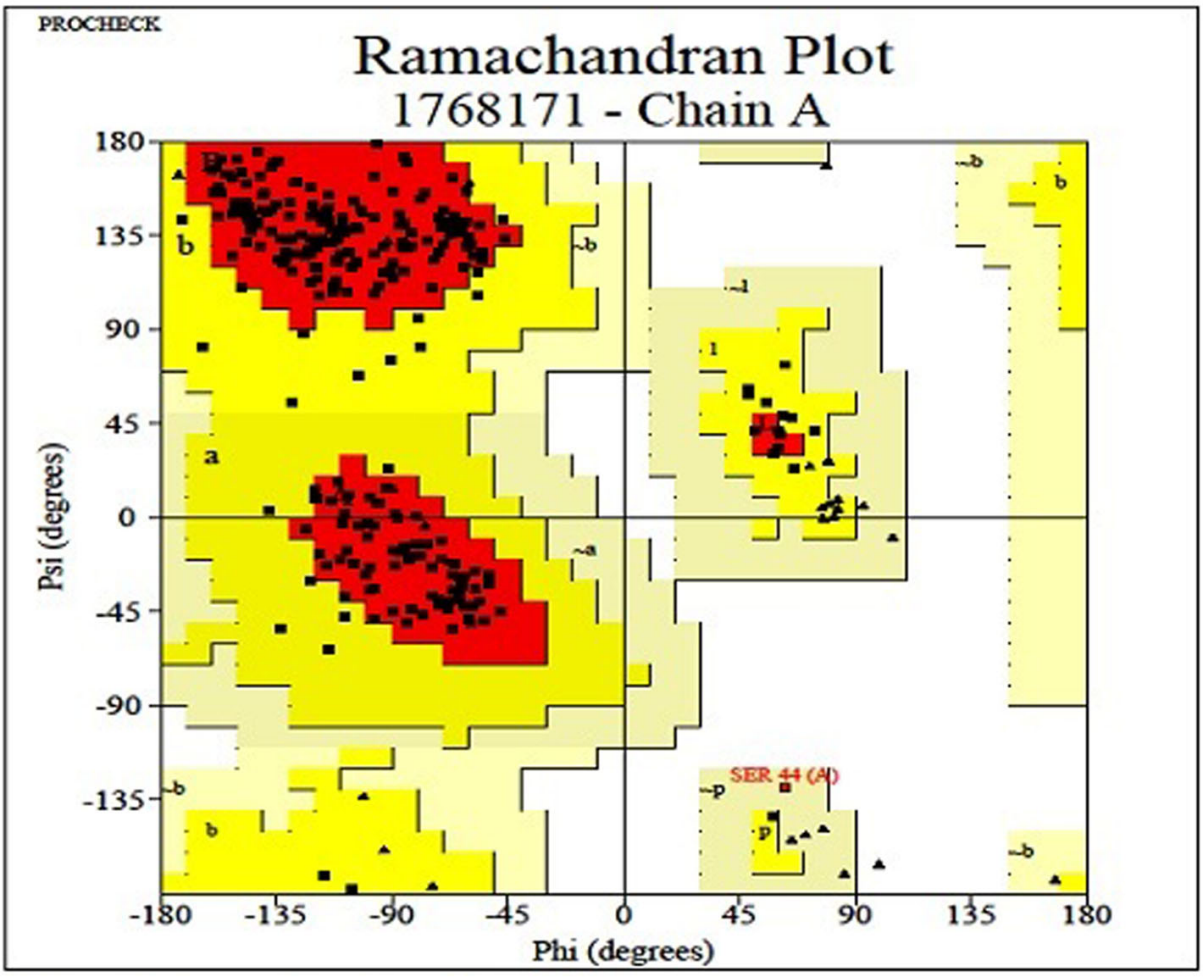
Ramachandran plot of STR

The modeling of the three-dimensional structure of the protein was performed by homology modeling program, Swiss Model. The final modeled structures were visualized by Swiss PDB Viewer that was shown in Fig. 1. For validation of generated structure, Ramachandran plot and PROVE plot were constructed. The Phi and PSi distribution of the Ramachandran map generated by non-glycine, non-proline residues were summarized in Table 6 and Fig. 2.

## Discussions

After retrieval of STR sequence, physicochemical properties were studied by Expasy’s ProtParam server (Table 2). The calculated isoelectric point (pI) will be useful because at pI, solubility is least and mobility in an electro-focusing system is zero. At pI, proteins were stable and compact. The computed isoelectric point value of P18417 was less than 7 (pI < 7) which indicates that this protein was considered acidic. The computed isoelectric point will be useful for developing a buffer system for purification by isoelectric focusing method. The instability index provides an estimate of the stability of protein in a test tube. A protein whose instability index is smaller than 40 is predicted as stable; a value above 40 predicts that the protein may be unstable [13]. The instability index value for P18417 (47.42) was found to be near 40 indicating that it was stable. The aliphatic index (AI) which is defined as the relative volume of a protein occupied by aliphatic side chains (A, V, I, and L) is regarded as a positive factor for the increase of thermal stability of globular proteins. Aliphatic index for the selected protein sequence was found to be 73.04. This higher value indicates the thermal stability of the protein. The grand average hydropathy (GRAVY) value for a peptide or protein is calculated as the sum of hydropathy values of all the amino acids, divided by the number of residues in the sequence. GRAVY indices of the selected protein was found to be − 285 which indicates. This lower value of GRAVY shows the possibility of better interaction with water.

The SOSUI server classifies the STR protein P18417 in the form of membrane protein. The transmembrane regions are rich in hydrophobic amino acids (Table 3).

Sequence of a particular cluster of residue types, which is variously known as a pattern, motif, signature, or fingerprint. These motifs, typically around 10 to 20 amino acids in length, arise because specific residues and regions thought or proved to be important to the biological function of a group of proteins are conserved in both structure and sequence during evolution [23]. Prosite analysis suggested the functionality of these proteins with profiles and patterns identified for characteristic functionality were represented in Table 4. In protein P18417, no motif was found in any range.

The secondary structure as predicted using SOPMA was represented in Table 5. The results show that random coil dominated among secondary structure elements followed by extended strand, alpha helix, and beta turns. Homology modeling is the method of choice for obtaining 3D coordinates of proteins. SWISS-MODEL workspace was an integrated web-based modeling expert system. For the STR, experimental protein structures were searched to identify suitable templates. On the basis of a sequence alignment between the STR and the template structure, a three-dimensional model for the STR was generated. STR catalyzes a Pictet-Spengler-type reaction and represents a novel six-bladed b-propeller fold in plant proteins [10]. STR from *R. serpentina* is a monomeric precursor protein with 344 amino acids that exhibits 100, 79, and 58% identity to STR1from *Rauvolfia mannii*, *C. roseus*, and *Ophior rhizapumila*, respectively. STR has been detected and biochemically characterized from cell suspension culture of apocynaceae plant *C. roseus* [24] and the Indian medicinal plant *Rauvolfia serpentina* [25] as well as from *Rubiaceae ophiorrhiza* [26]. Three-dimensional structures are predicted for proteins where such data is unavailable. There is lack of experimental structures for this protein considered. Swiss model was used to generate the homology model for STR that has not been reported in literature yet. The protein-ligand interaction was found to be very important. 3D structure enables us to understand the binding specificities of ligands with protein.

### Validation of STR model

Homology modeling was the most accurate computational method to generate reliable structural models. It was used in many biological applications. Model quality assessment tools were used to estimate the reliability of the models. The stereochemical quality of the predicted model and accuracy of the protein model was evaluated after the refinement process using Ramachandran map calculations computed with the PROCHECK program. The assessment of the predicted model generated by Swiss model was shown in Fig. 2. The main chain parameters plotted are Ramachandran plot quality, peptide bond planarity, bad non-bonded interactions, main chain hydrogen bond energy, C-alpha chirality, and overall G factor. In the Ramachandran plot analysis, the residues were classified according to its regions in the quadrangle. The red regions in the graph indicate the most allowed regions whereas the yellow regions represent allowed regions. Glycine is represented by triangles and other residues are represented by squares. The result revealed that the modeled structure for P18417 has 88.8% residue in allowed region. Such figures assigned by Ramachandran plot represent a good quality of the predicted models.

PROVE plot was used to calculate the atoms in the modeled structure of strictosidine synthase, which shows the particular result in Table 7. The RMS *Z* score was found to be more than 1 that also supports good quality of model structure.

## Conclusion

In this study, STR that plays an important role in alkaloid biosynthesis of *C. roseus* was selected. Physicochemical characterization was performed by computing theoretical isoelectric point (pI), molecular weight, total number of positive and negative residues, extinction coefficient, instability index, aliphatic index, and grand average hydropathy (GRAVY). Functional analysis of this protein was performed by SOSUI server which predicted the transmembrane helix. Secondary structure analysis revealed that Alpha helix dominated among secondary structure elements followed by random coil, extended strand, and beta turns. The modeling of the three-dimensional structure of the Strictosidine synthase (P18417) was performed by Swiss model. The model was validated using protein structure checking tools PROCHECK and PROVE.

## Data Availability

Not applicable

## Abbreviations

*C. roseus*: *Catharanthus roseus*
STR: Strictosidine synthase
GRAVY: Grand average hydropathy
AI: Aliphatic index
II: Instability index
pI: Isoelectric point
SOPMA: Self-Optimized Prediction Method with Alignment
